# Supplementary material for: LADON, a Natural Antisense Transcript of NODAL, Promotes Tumour Progression and Metastasis in Melanoma
Source: Noncoding RNA. 2023 Nov 15;9(6):71. doi: 10.3390/ncrna9060071 (PMC10661258; doi:10.3390/ncrna9060071)
Supplement: Supplementary file 1 [file ncrna-09-00071-s001.zip › ncrna-2683212-supplementary figure.pdf]

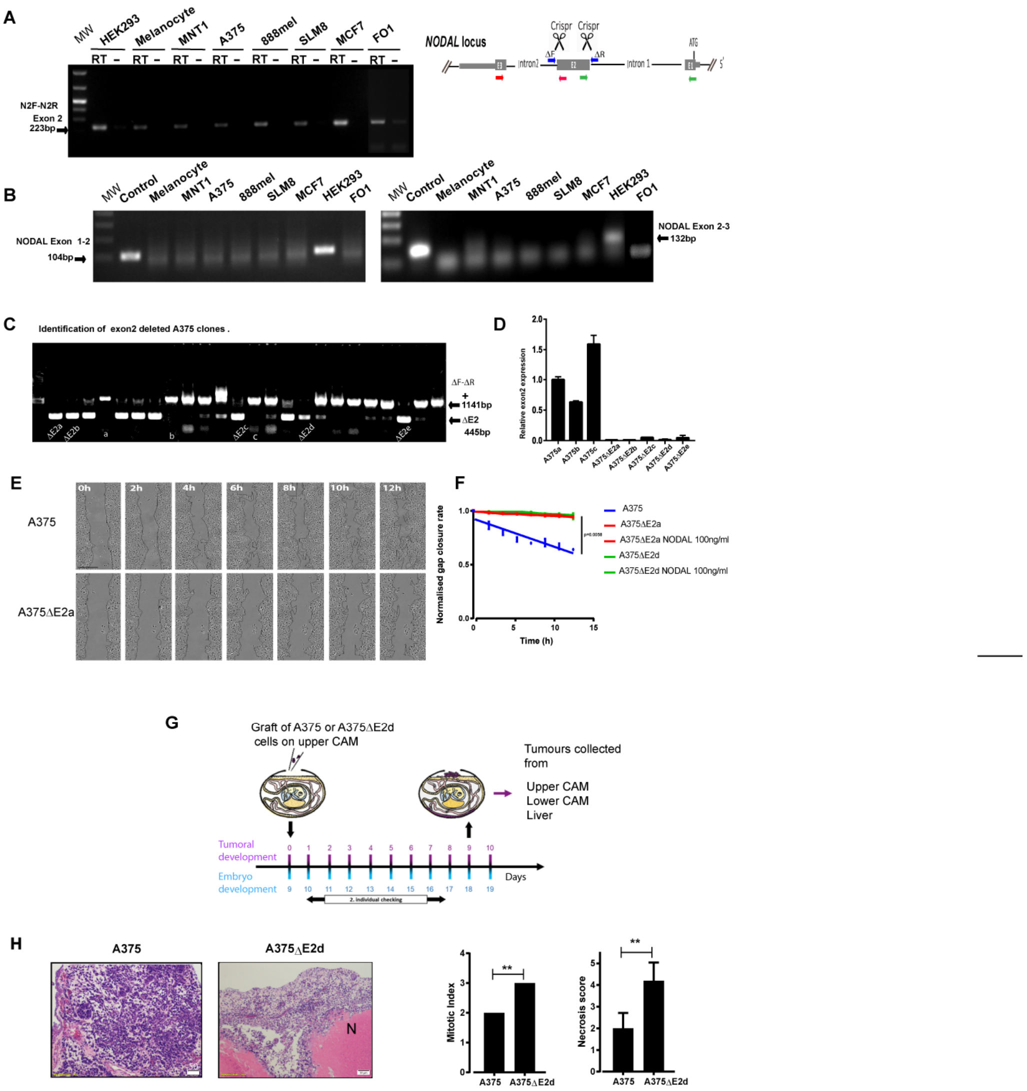

Figure S1: Characterisation of A375ΔE2 clones (**A**) Left panel: RT-PCR with the primers N2R and N2F to detect the transcription of *NODAL* exon2 in a panel of cell lines: embryonic kidney (HEK293), melanocytes, non-metastatic melanoma (MNT1), metastatic melanoma (A375, 888mel, SLM8, FO1), breast cancer (MCF7). The expected 223bp band is detected in all reverse-transcribed samples (noted RT), but not in samples without reverse transcriptase (noted -). Right panel: Schematic of the human *NODAL* locus with its 3 exons (E1 to E3), and the primer pairs used to detect its expression. (**B**) Left panel: RT-PCR with primers spanning the *NODAL* exon 1-2 boundary in the same cell lines detected the expected 104bp band only in the HEK293 cell line and in the positive control, which is a plasmid containing a cDNA of *NODAL*. Right panel: RT-PCR with primers spanning the *NODAL* exon 2-3 boundary in the same cell lines detected the expected 132bp band only in the HEK293 cell line and in the positive control, which is a plasmid containing a cDNA of *NODAL*. (**C**) PCR amplification with the ΔF-ΔR primer pair of the genomic region targeted by the CRIPSR/Cas9-mediated deletion of *NODAL* exon2 in A375 cells. In the 24 independent clones analysed here (out of a total of 96), amplification of the intact locus yields a 1141nt-long band, while that of the deleted one (ΔE2) yields a 445nt-long band. + and Δ indicate examples of unmodified and exon2-deleted clones. (**D**) RT-qPCR of *NODAL* exon2 with the N2F/N2R primer pair detects the presence of a transcript in 3 A375 parental clones (A375a-c), but not in the 5 independent mutant clones A375ΔE2a to e. (**E**)Representative images, acquired at *t*=0, 2, 4, 6, 8, 10 and 12h, of scratch-wound healing assays performed with A375 and A375ΔE2a cells. **(F)** Normalized gap closure rates of A375, A375ΔE2a and A375ΔE2d cells treated with or without recombinant NODAL. The *p*-value was calculated by a two-way ANOVA test. **(G)** Schematic of the CAM assay. An inoculum of cells is deposited onto the upper CAM of each chicken

embryo on day E9. On day E18 all tumours on the upper CAM are weighed, and the lower CAM and liver are collected to quantify the presence of metastatic cells via qPCR detection of human Alu sequences. **(H**)Left panel: Higher magnification views of the tumour sections shown in Fig.1D. N, necrosis. Scale bar=50 µm. Right panel: Mitotic index, determined by counting the number of mitoses in 10 non-overlapping, non-necrotic fields observed at 40X magnification on sections of A375 and A375ΔE2d-derived tumours (*n*=5 each). 0: no mitotic figure; 1: 1 mitotic figure; 2: 2 to 3 mitotic figures; 3: more than 3 mitotic figures. Necrosis score, determined by measuring the extent of necrosis on 10 non-overlapping fields observed at 10X magnification on the same sections as before. 0: no necrosis; 1: small foci of necrosis or scattered; 2: necrosis in <50% of the tumour area; 3: necrosis in 50-75% of the tumour area; 4: necrosis in 75-90% of the tumour area; 5: necrosis in >90% of the tumour area. ** $p<0.01$

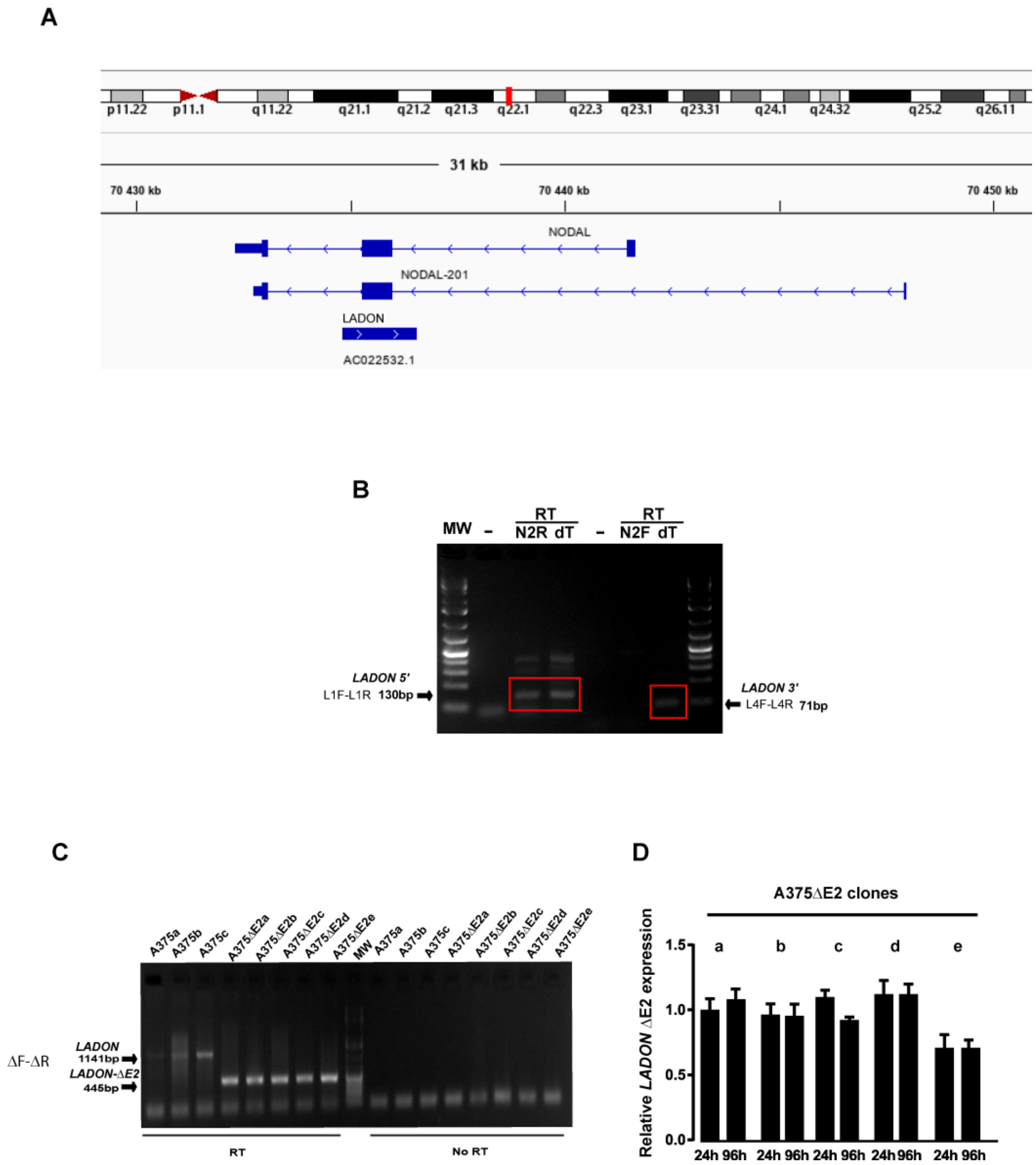

FigureS2: Characterisation of *LADON* expression in unmodified and mutant A375 cells
(A) Human NODAL locus, adapted from GRCh38 database. **(B)** PCR amplification of reverse-

transcribed total A375 RNA yields bands for *LADON* from cDNAs primed with an oligo dT or the reverse primer N2R, but not from cDNAs primed with the forward primer N2F. No band is amplified in samples without reverse transcriptase (noted -). (**C**) RT-PCR using primers spanning the entire exon2 detects the presence of the *LADON* transcript in A375 clones a to c, and the *LADON-ΔE2* transcript, a truncated version of *LADON*, in the exon2-deleted clones A375ΔE2a to e. (**D**) RT-qPCR analysis of *LADON-ΔE2* expression in five independent A375ΔE2 clones detects no increase over the course of the culture.

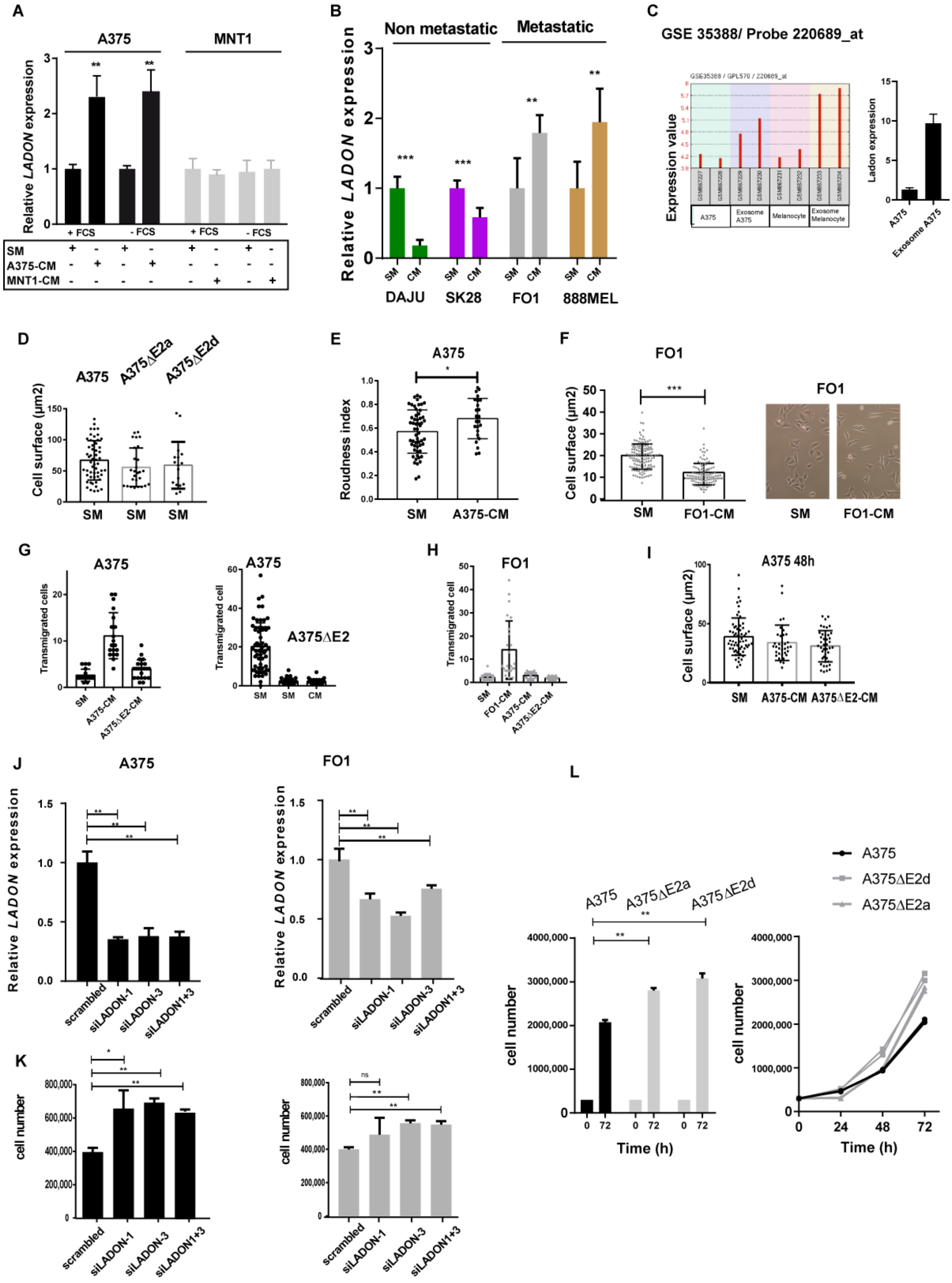

Figure S3: Causes and consequences of changes in *LADON* expression.(**A**) RT-qPCR analysis of *LADON* expression in A375 and MNT1 cells cultured for 24h in standard culture medium (SM), in A375-conditioned medium (A375-CM) or in MNT1- conditioned medium (MNT1-CM), with or without fetal calf serum (FCS). Histograms display mean values ± SD from a minimum of three independent replicates. *p*-values were calculated by Student's t test * $p < 0.05$.
(**B**) RT-qPCR analysis of *LADON* expression in non-metastatic (DAJU, SK28) and metastatic (FO1, 888MEL) melanoma cell lines cultured for 24h in SM or in their own CM. ** $p < 0.01$, **** $p < 0.001$
(**C**) Right panel; *LADON* expression levels measured in melanoma cells (A375), in normal melanocyte (HEMa), and in their exosomes (GSE35388 dataset on the Affymetrix Human Genome U133 Plus 2.0 Array analysis with the 220689_at probe set). Left panel; *LADON* expression in A375 and purified exosomes. (**D**) The histogram shows cell surface measurements of A375, A375ΔE2a and A375ΔE2d cells cultured for 24h in SM. (**E**) The histogram shows roundness index measurements of A375 cells cultured for 24h in SM or A375-CM. , * $p < 0.05$ (**F**)The histogram shows cell surface measurements of FO1 cells cultured for 24h in SM or FO1- CM. **** $p < 0.001$. (**G**)Transmigrated cell counts for the data presented in Fig 3G. (**H**)Transmigrated cell counts for the data presented in Fig 3H. (**I**)The histogram shows cell surface measurements of A375 cells cultured for 48h in SM, A375-CM or A375ΔE2-CM
(**J**) RT-qPCR analysis of LADON expression in A375 or FO1 cells treated with the siRNAs scrambled, siLADON-1, siLADON-3, or siLADON-1 and -3. Values are normalized to that obtained with the scrambled siRNA, which is set to 1. ** $p < 0.01$. (**K**) Comparison of cell counts in A375 or FO1 cells cultured for 72h in the presence of siRNAs scrambled, siLADON-1, siLADON-3, or siLADON-1 and -3. * $p < 0.05$, ** $p < 0.01$, ns non significant. (**L**) Left panel: Comparison of cell counts in A375, A375ΔE2a, A375ΔE2d, A375ΔE2dS1 and A375ΔE2dS2 cells after 72h culture. A375ΔE2dS1 and A375ΔE2dS2 are clones of A375ΔE2d that have stably integrated a *LADON*-expressing construct. Right panel: Graph showing the same data including additional time points at 24 and 48h., ** $p < 0.01$.
