# Supplementary material for: LADON, a Natural Antisense Transcript of NODAL, Promotes Tumour Progression and Metastasis in Melanoma
Source: Noncoding RNA. 2023 Nov 15;9(6):71. doi: 10.3390/ncrna9060071 (PMC10661258; doi:10.3390/ncrna9060071)
Supplement: Supplementary file 1 [file ncrna-09-00071-s001.zip › Table S2.pdf]

Table S2.The primers used for qRT-PCR, CRISPR/CAS9 genome editing and siRNA.

Amplicon	Sequence (5′-3′)	Amplicon size
CRISPR/CAS9 Exon 2 Deletion 5′	F- ATCCACTGCCACATCTGGGTGTTTT<br>R- ACCCAGATGTGGCAGTGGATCGGTG	
CRISPR/CAS9 Exon 2 Deletion3′	F- GACCAACCATGCATACATCCGTTTT<br>R- GGATGTATGCATGGTTGGTCCGGTG	
ladon ΔE2detection	F- ATCTTGGAAGGGGGACTGGAA<br>R- TCCTGGTACATTGGAGGTGCTT	1141- 445
Nodal Exon2	N2F- CTGCTTAGAGCGGTTTCAGATG<br>N2R- CGAGAGGTTGGAGTAGAGCATAA	223
Nodal Exon 1-2	F- CAGACATCATCCGCAGCCTA<br>R- TGCCAGATCCTCTTGTTGGC	104
Nodal Exon 2-3	F- AGCAGTACAACGCCTATCGC<br>R- AACAAGTGGAAGGGACTCGG	132
LADON 5′	L1F- CAAAGCTAGAGCCCTGTCCC<br>L1R- AGGGCGAGTGTCCTAATCCT	130
LADON 5′	L2F- TGGTACATTGGAGGTGCTTG<br>L2R- TGAGGGCGAGTGTCCTAATC	188
LADON 3′	L4F- AAGCAAACGTCCAGTTCTGCC<br>L4R- TCACCCTCCTTCCTTCTTGGT	71
RPL13	F- GGAAGTACCAGGCAGTGACAGC<br>R- CTTCTCGGCCTGTTTCCGTAG	110
siRNA LADON	1) CAGCUAAUGAGGAGUCAAAUU<br>2) CAUUGGAGGUGCUUGAGU<br>3) ACAGGAACAUAGUCAUUUAUU	
probe set 220689_at	CTCAGTCAGTGTCACAACCACCATA<br>CAACCACCATAGCTCTTGCTGGAGG<br>CTTGCTGGAGGCCTTACCTTATATA<br>GATTTTCGAGTCCCTTTTAGAGATG<br>AGTAAACCTGGATTTGCCCAACTGC<br>AGCCCAGACTCCGAATTACTGTTCT<br>GTCCCCCTTCCAAGATAACTTGCAC<br>CTTGCACAGCCAGAGGACAGTCAGT<br>AAATGTGATGAGTTGCATCTCCCCC<br>CTCCTCCCATACATGAAGTCTGGCC<br>CCCAGCCACTCGAGAATCATTTGAA	
